# Biofilm reduction potential of 0.02% polyhexanide irrigation solution in several types of urethral catheters

**DOI:** 10.1186/s12894-021-00826-3

**Published:** 2021-04-09

**Authors:** Florian H. H. Brill, Julia Hambach, Christian Utpatel, Diana C. Mogrovejo, Henrik Gabriel, Jan-Hendrik Klock, Joerg Steinmann, Andreas Arndt

**Affiliations:** 1 Dr. Brill + Partner GmbH Institute for Hygiene and Microbiology, Stiegstück 34, 22339 Hamburg, Germany; 2 Institute of Immunology, University Medical Center Hamburg-Eppendorf, Hamburg, Germany; 3 Molecular and Experimental Mycobacteriology, Research Center Borstel – Leibniz Lung Center, Borstel, Germany; 4 Klinikum Nürnberg, Institute of Clinical Hygiene, Medical Microbiology and Infectiology, Paracelsus Medical University, Nuremberg, Germany; 5 Department of Research and Development, B. Braun Medical Ltd., Sempach, Switzerland

**Keywords:** Bacterial decolonization, Biofilm, Polyhexanide, Urinary catheter, Urinary tract infection, Transurethral catheter

## Abstract

**Background:**

Long-term use of urethral catheters is associated with high risk of urinary tract infection (UTI) and blockage. Microbial biofilms are a common cause of catheter blockage, reducing their lifetime and significantly increasing morbidity of UTIs. A 0.02% polyhexanide irrigation solution developed for routine mechanical rinsing shows potential for bacterial decolonization of urethral catheters and has the potential to reduce or prevent biofilm formation.

**Methods:**

Using an in vitro assay with standard market-leading types of catheters artificially contaminated with clinically relevant bacteria, assays were carried out to evaluate the biofilm reduction and prevention potential of a 0.02% polyhexanide solution versus no intervention (standard approach) and irrigation with saline solution (NaCl 0.9%). The efficiency of decolonization was measured through microbial plate count and membrane filtration.

**Results:**

Irrigation using a 0.02% polyhexanide solution is suitable for the decolonization of a variety of transurethral catheters. The effect observed is significant compared to irrigation with 0.9% saline solution (*p* = 0.002) or no treatment (*p* = 0.011). No significant difference was found between irrigation with 0.9% saline solution and no treatment (*p* = 0.74).

**Conclusions:**

A 0.02% polyhexanide solution is able to reduce bacterial biofilm from catheters artificially contaminated with clinically relevant bacteria in vitro. The data shows a reduction of the viability of thick bacterial biofilms in a variety of commercially available urinary catheters made from silicone, latex-free silicone, hydrogel-coated silicone and PVC. Further research is required to evaluate the long-term tolerability and efficacy of polyhexanide in clinical practice.

**Supplementary Information:**

The online version contains supplementary material available at 10.1186/s12894-021-00826-3.

## Background

Urinary tract infections are among the most common nosocomial infections. In Germany, for instance, it was estimated that approximately 155,000 nosocomial urinary tract infections occur every year, and the majority of these cases are catheter-associated [1]. Catheters, as many inserted medical devices, are heavily prone to microbial biofilm formation [2]. A variety of pathogens are able to colonize catheters: commensal species of bacteria from the gastrointestinal tract or ascending from the urethra, or bacteria transferred from the insertion site [2, 3].

Pathogens such as *Escherichia coli*, *Enterobacter* spp., *Pseudomonas* spp., *Enterococcus* spp., *Staphylococcus aureus*, coagulase-negative staphylococci and yeasts are common causes of urinary tract infections and catheter blockage [3, 4] and the most commonly reported species forming biofilms on urethral catheters are *Candida* spp., *Pseudomonas aeruginosa*, *Proteus mirabilis, E. faecalis*, and *S. aureus* [2, 3]. Scanning electron microscopy performed on biofilms formed on indwelling catheters has shown depths ranging from 3 to 490 µm and up to 400 visible bacterial cells deep [5].

In a biofilm, microbes are attached to the catheter surfaces in a manner that prevents their removal with gentle rinsing and would require mechanical removal. In fact, biofilms formed in catheters often lead to catheter encrustation and obstruction [5]. Biofilms in catheters have important implications for health as antibiotics are rarely able to penetrate the superficial layers of the biofilm, complicating treatment [5]. Moreover, microbial biofilms are known to be up to 1500 times more resistant to antibiotic therapy compared to planktonic, free-living bacteria [3, 6]. Biofilms on catheters can lead to significant complications and unfavorable outcomes for the patients’ health [3] and for this reason, the development of effective methods and compounds for the prevention of biofilm formation or their reduction is of great importance [2, 3, 7].

Polyhexanide (polyhexamethylene biguanide or PHMB) is a polymer frequently used as an antiseptic with broad antibacterial activity, good tissue tolerability and, to date, shows no development of bacterial resistance [8]. Polyhexanide has been used for mechanical rinsing and removal of biofilms across a range of applications [4, 7, 9]. In this study, we investigated the potential of a polyhexanide solution to reduce and prevent biofilm formation under in vitro conditions in a variety of artificially colonized catheters.

## Methods

All experiments were performed using a mixed culture of the following bacterial strains: *Escherichia coli* (ATCC® 11229), *Proteus mirabilis* (ATCC® 14153, DSM 778) and *Staphylococcus* *aureus* (ATCC® 6538). An overnight culture plate in nutrient agar (OXOID, Germany) of each bacterium was washed away in 10 mL NaCl peptone and transferred to a sterile flask with glass beads. This suspension was homogenized for 2 min at 1500 rpm on a mechanical shaker and adjusted to 10^9^ CFU per mL using standard plate count methods (data not shown).

Five different types of catheters were used, as shown in Table 1. These different catheters were selected to cover a broad range of commercially available products and observe the performance of the treatments in all of them. Due to its popularity as biomaterial [10–12], 4 catheters made of silicone out of the 5 were used in this study. Of these, Catheter A is the only one to have a hydrogel coating.

**Table 1:** Characteristics of the catheters used in this study

Reference for experiments	Specifications
Catheter A	2-way Foley catheter, latex-free silicone, Balloon 30 cc, 18Ch, hydrogel coating
Catheter B	Straight whistle tip catheter, silicone, 40 cm, 18Ch
Catheter C	Bladder catheter without balloon, PVC, 37 cm, 18Ch, latex-free
Catheter D	Transurethral Foley catheter, 2-way, latex-free silicone, 41 cm, 18Ch
Catheter E	Transurethral Foley Nelaton balloon catheter, latex-free silicone, 41 cm, 18Ch

Thirty (30) catheters of each type were used for the decolonization test. The catheters were incubated with 5 ml of the mixed bacterial suspension for 4 h at 37 °C, after which the catheters were irrigated 2 × 400 ml of an organic load suspension (0.3% bovine albumin + 3.0% urea, reagents from Carl Roth Germany) per day to simulate the process of contamination with urine and organic materials. After 72 h, ten of the catheters were irrigated with 100 mL Uro-Tainer® 0.02% PHMB (B. Braun Medical, Switzerland) with 5 min exposure time, ten catheters were irrigated with 100 mL Uro-Tainer® 0.9% NaCl (B. Braun Medical, Switzerland) with an exposure time of 5 min and 10 catheters were not treated (controls).

After treatment, the microbial count was determined by irrigation of the catheters with 100 ml of a TLH-SDS neutralizer solution (0.1% polysorbat 80, 0.1% g/L lecithin, 0.1% histidine, 0.2% SDS, all reagents from Carl Roth Germany) and membrane filtration (0.45 µm pore size, MF-Millipore USA) of 50 mL via serial dilution method on trypticase soybean agar (TSA, OXOID Germany). No measurements of pH were made for the rinsed filtrates as the slightly acidic pH of the Uro-Tainer® 0.02% PHMB (pH at 20 °C of 5.5) was neutralized with the use of the TLH-SDS solution. We anticipated that the pH of the filtrates did not modify the pH of the culture media used in this study and therefore did not affect the growth of the surviving bacteria.

In addition, all catheters were cut and the material in the lumen was extracted with a sterile cotton swab. The swab was suspended in 0.9% NaCl solution (Carl Roth Germany) and the microbial count was determined via serial dilution method on TSA plates.

All nutrient media were incubated at 37 °C for 2 days. Mean values of microbial count (log_10_ CFU) after the different treatments were calculated as well as the reduction factors for the Uro-Tainer® 0.02% PHMB solution compared to Uro-Tainer® 0.9% NaCl and no treatment. Statistical analyses were done using the two-tailed Student’s ‘*t*’ test and *p* values of (* ≤ 0.05) were considered as significant.

## Results

**Fig. 1:**
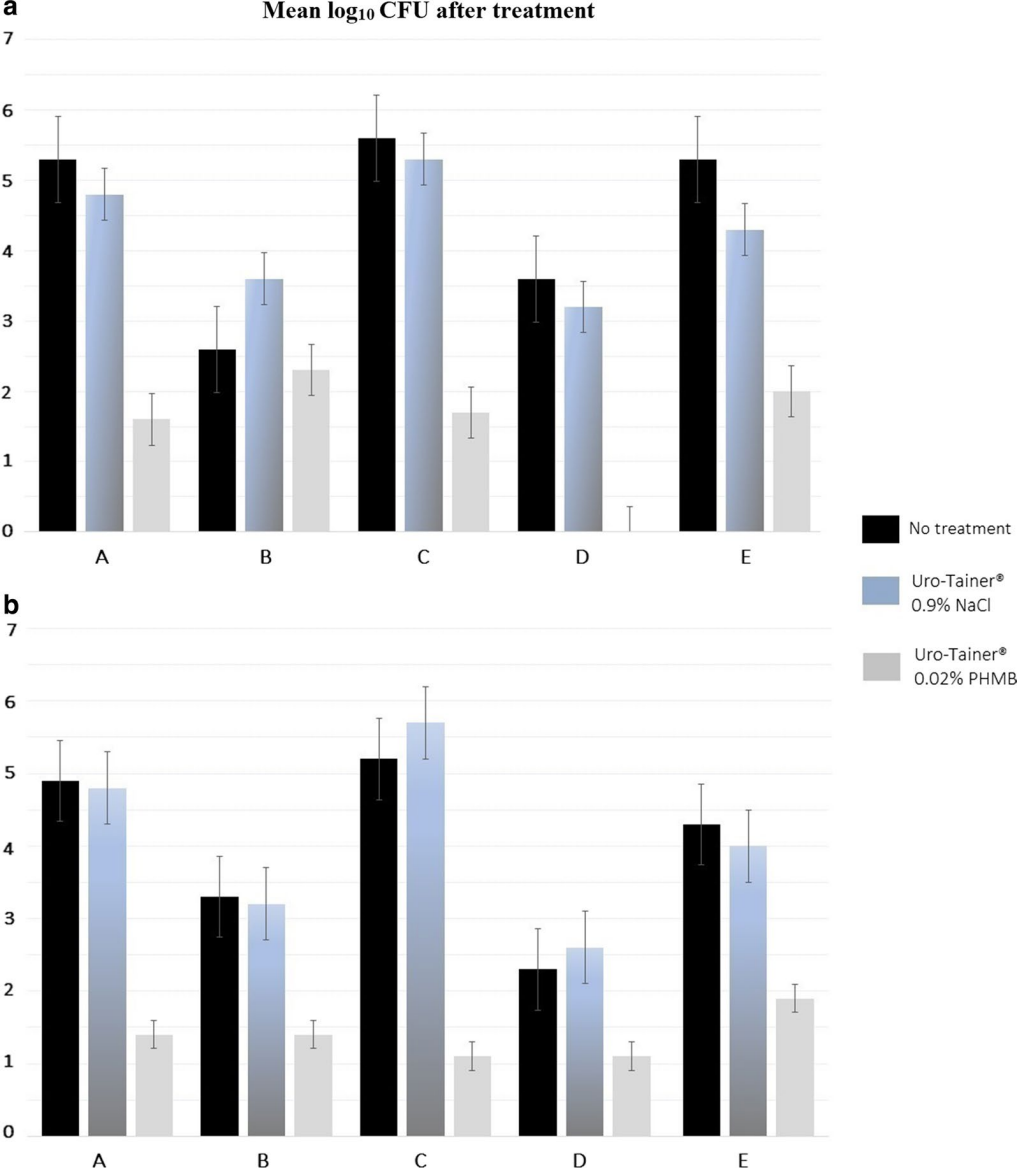
Cell counts after filtration of **a** rinsing solutions and **b** after swab sampling of catheters treated with different irrigation solutions. The values of log_10_ CFU are shown as the mean of 10 parallel replications

We observed that treatment with the Uro-Tainer® 0.02% PHMB solution effectively reduced the biofilms artificially formed in the different catheter types as measured in after rinsing/membrane filtration (Fig. 1a) as well as in the swab samples (Fig. 1b). In general, the effect of irrigation with Uro-Tainer® 0.02% PHMB solution is superior compared to the untreated catheters (*p* = 0.011) as well as compared to those treated with Uro-Tainer® 0.9% NaCl solution (*p* = 0.002) while there was no significant effect of irrigation with 0.9% NaCl compared with no treatment at all (*p* = 0.74) (Additional file 1: Table S1). Reductions factors (Table 2) ranged from 0.3 in the case of the type B catheter with no treatment to 4.6 for the type C catheter treated with Uro-Tainer® 0.9% NaCl.

**Table 2:** Biocidal activity of Uro-Tainer® 0.02% PHMB compared to Uro-Tainer® 0.9% NaCl and no treatment represented by Reduction factors of log_10_ CFU

Catheter	Reduction factors using Uro-Tainer® 0.02% PHMB compared to			
	Uro-Tainer® 0.9% NaCl	No treatment	Uro-Tainer® 0.9% NaCl	No treatment
	Cell counts after filtration		Cell counts after swab sampling	
Catheter A	3.2	3.7	3.4	3.5
Catheter B	1.3	0.3	1.8	1.9
Catheter C	3.6	3.9	4.6	4.1
Catheter D	1.2	1.6	1.5	1.2
Catheter E	2.3	3.3	2.1	2.4

## Discussion

In natural, clinical, and industrial environments, the formation of biofilms is a basic microbial survival strategy. Medical devices such as suprapubic and indwelling catheters used in clinical settings are frequently colonized by biofilms of a variety of microbial species with detrimental consequences for the patients [13]. Research is focused on decolonization of urethral catheters via treatment with a range of systemic antibiotic regimens [14] even though antibiotic resistance and drug adsorption are heavily modified in biofilms [3, 4, 13]. In fact, antimicrobial compounds are usually not able to penetrate the full depth of the microbial biofilms, reducing the available options for effective therapy [15]. Treatment is further complicated by the fact that biofilms are frequently composed of a variety of species, as demonstrated from urine samples of colonized-catheters [16].

Besides antimicrobial therapy, the treatment of infections caused by bacterial biofilms in catheters requires the removal of the bacteria attached to the devices or at least, a noticeable reduction of the bacterial load [17]. Alternative methods and compounds for reduction and prevention of biofilms in catheters are necessary as altered catheter surfaces have proven ineffective at inhibiting microbial attachment [3, 15, 18].

In this regard, polyhexanide is considered one of the “most promising substances available” for clinical applications [19]. Mechanical rinsing with 0.9% NaCl solution and a 0.02% solution of polyhexanide has been observed to significantly and consistently reduce bacterial colonization, providing an effective, non-systemic approach to biofilm formation on urinary catheters [4]. Other studies have also confirmed the antiseptic efficacy and antibacterial effect of polyhexanide in the treatment of skin wounds, as an ingredient in mouthwash solutions or as a supplement of cleansing solutions [19]. Furthermore, observational studies in patients with indwelling catheters in which the Uro-Tainer® 0.02% PHMB solution was used for rinsing, showed no serious adverse events for the patients [20].

Here, the reduction of biofilms in artificially colonized commercially available catheters using Uro-Tainer® 0.02% PHMB solution was tested in an in vitro assay. The study confirms the efficacy of the solution on a range of catheters types. Our results showed that rinsing the catheters reduces colonization and the treatment with polyhexanide was more effective compared to rinsing with saline solution or no rinsing at all (*p* = 0.002 and *p* = 0.011, respectively), in all 5 types of catheters tested. These results go in accordance to previous reports of the usefulness of polyhexanide [19, 21–23] and confirms the efficacy of a 0.02% polyhexanide solution to reduce and prevent the formation of biofilms in catheter systems in vitro [4].

The formation of biofilms in urethral catheters is aided by the deposition of organic molecules from the urinary components, such as proteins and electrolytes [16]. The inert materials of catheters make them susceptible to microbial colonization [16], as they cannot produce the immunological response triggered in the mucosa of living tissue responsible for neutralizing colonizing microbes. While some bacteria have proven to adhere less to certain materials, as is the case of *E. coli* and *Klebsiella pneumoniae* in siliconized-rubber catheters [16, 24], with enough time the microflora inevitably finds a way to colonize the catheter. Thus, the characteristics of the catheter material is critical for both the biocompatibility of the device as well as for reducing and/or avoiding bacterial adhesion [17]. The interactions between bacteria and the polymers in the biomaterials are mediated by forces such as hydrophobic interactions between surfaces [25] or fimbrial interactions [10]. The initial adherence seems to depend mainly on the catheter biomaterial and it is later influenced in vivo by other factors such as the presence of proteins, fluids and host cells [25]. Moreover, the composition of already-formed microbial biofilms does not appear to be influenced by the biomaterial [26].

In our study, the result of the decolonization treatment with 0.02% PHMB was significantly different from the treatment with 0.9% NaCl in all the catheters tested, confirming the efficacy of polyhexanide in a variety of biomaterials, such as silicone, latex-free silicone, hydrogel-coated silicone and PVC besides its efficacy already proven in polyurethane catheters [4]. We observed a similar behavior between the hydrogel-coated silicon catheter A, the PVC catheter C and the silicone catheter E (Fig. 1).

Silicone is the most popular material in catheters [10–12] followed by other materials like PVC, polyurethane and latex. Hydrogel-coated catheters, while useful to decrease the mucosal trauma derived from catheter use, are comparable to other catheters in the formation of biofilm [27]. A review of the literature suggests that not one material is significantly better than another in preventing the encrustation of long-term inserted medical devices [28], although smooth non-sticky materials such as silicone appear to behave more favorably while latex-based catheters seem to favor biofilm formation more often due to their uneven surfaces and irregularities [27]. On the other hand, there is insufficient scientific evidence for a decreased frequency of bacteriuria or catheter-associated UTI due to obstruction in silicone compared to latex catheters [27]. Hence the importance of methodologies that, besides improvements on catheter material, allow the reduction of the bacterial load in nascent biofilms.

Some materials contain additives meant to improve the properties of the device [17] but have been shown to bloom to the surface with natural aging where they are metabolized by organisms and facilitate their attachment, e.g. polyurethane and PVC [17, 29]. Combinations of materials, such as polyurethane and polycarbonate, seem to provide a solution for blooming [30]. Other materials, such as PVC and siliconized latex, appear to intrinsically favor the adherence of bacteria [25] even allowing growth in the absence of conventional nutrients [31]. In this study, the specifications of Catheter B did not indicate whether latex was used for its fabrication and perhaps the presence of this biomaterial could explain the lowest reduction factor observed (RF = 0.3) when the treatment with 0.02% PHMB was compared to no treatment at all (Table 2). However, we also observed higher reduction factors for catheter C, the only one made of PVC, along with the hydrophobic, hydrogel-coated catheter A, confirming that many other factors influence bacterial adherence to catheters besides biomaterial [25].

This study provides evidence of the efficacy of Uro-Tainer® 0.02% PHMB to reduce biofilms possibly through a combination of mechanical and antibacterial effects and independently of the catheter characteristics. Our results show significant reductions of the log_10_ CFU in all tested catheters. Such efficacy is of great importance as catheter material generally does not diminish the ability of microbes to form biofilms within them, except in the setting of short-term catheterization [16].

Additional research is recommended to investigate whether the presented results can be transferred into practice and lead to a reduction in urinary tract infections in clinical settings. Parameters such as the pH of patients’ urine as well as retrograde bacterial growth into the bladder could be investigated further to determine their effect on the efficacy of polyhexanide.

## Conclusion

Our experiments show that rinsing with Uro-Tainer® 0.02% PHMB on artificially colonized silicone catheters, latex-free silicone catheters, latex-free PVC catheters and latex-free hydrophobic (hydrogel-coated) silicone catheters, is significantly more effective compared to rinsing with saline solution or no rinsing at all.

The use of a polyhexanide solution constitutes a strategy for the reduction of the viability of thick bacterial biofilms in a variety of commercially available urinary catheters.

## Supplementary Information

**Additional file 1: Table S1**. Cell counts of rinsing solutions and of swab samples after different treatments. The values of log10 CFU shown are mean of 10 parallel replications.

## Data Availability

The data supporting the conclusions of this article is included within the article (and its Additional file 1: Table S1). Additional raw datasets used and/or analyzed during the current study are available from the corresponding author on reasonable request.

## Abbreviations

UTI: Urinary tract infection
PHMB: Polyhexanide (polyhexamethylene biguanide)
spp.: Species
mL: Milliliter
nm: Nanometer
µm: Micrometer
h: Hour(s)
min: Minutes
g/L: Grams per liter
rpm: Revolutions per minute
