# Supplementary material for: Biofilm reduction potential of 0.02% polyhexanide irrigation solution in several types of urethral catheters
Source: BMC Urol. 2021 Apr 9;21:58. doi: 10.1186/s12894-021-00826-3 (PMC8034122; doi:10.1186/s12894-021-00826-3)
Supplement: Supplementary file 1 — Additional file 1: Table S1. Cell counts of rinsing solutions and of swab samples after different treatments. The values of log10 CFU shown are mean of 10 parallel replications. [file 12894_2021_826_MOESM1_ESM.docx]

**Manuscript ID:** BURO-D-20-00497R2

**Title:** Biofilm prevention potential of 0.02% polyhexanide irrigation solution in urethral catheters under practice-like in vitro conditions

**Authors:** Florian H. H. Brill; Julia Hambach; Christian Utpatel; Diana Mogrovejo; Henrik Gabriel; Jan-Hendrik Klock; Joerg Steinmann; Andreas Arndt

**Supplementary material**

**TABLE S1**

**Table S1.** Cell counts of rinsing solutions and of swab samples after different treatments. The values of log_10_ CFU shown are mean of 10 parallel replications.

| **Type of catheter** | **Mean** **log_10_ CFU after treatment** | | | | | |
| --- | --- | --- | --- | --- | --- | --- |
|  | **Cell counts after filtration** | | | **Cell counts after swab sampling** | | |
|  | **Uro-Tainer®**  [**0.02 % PHMB**](#_bookmark3) | **Uro-Tainer®**  **0.9 % NaCI** | **no treatment** | **Uro-Tainer®**  [**0.02 % PHMB**](#_bookmark3) | **Uro-Tainer®**  **0.9 % NaCI** | **no treatment** |
| **Catheter A**  **(Ref. 176818)** | 1.6 | 4.8 | 5.3 | 1.4 | 4.8 | 4.9 |
| **Catheter B**  **(Ref. AD6518)** | 2.3 | 3.6 | 2.6 | 1.4 | 3.2 | 3.3 |
| **Catheter C**  **(Ref. 250100)** | 1.7 | 5.3 | 5.6 | 1.1 | 5.7 | 5.2 |
| **Catheter D**  **(Ref. 171305)** | 2 0 | 3.2 | 3.6 | ≤ 1.1 | 2.6 | 2.3 |
| **Catheter F**  **(Ref. 04563182)** | 2.0 | 4.3 | 5.3 | 1.9 | 4.0 | 4.3 |
| **Average** | 1.92 | 4.24 | 4.48 | 1.38 | 4.06 | 4 |
| **Standard deviation** | 0.277 | 0.856 | 1.314 | 0.327 | 1.236 | 1.196 |
